# Enhancing Aerosol Mitigation in Medical Procedures: A CFD-Informed Respiratory Barrier Enclosure

**DOI:** 10.3390/bioengineering11111104

**Published:** 2024-11-01

**Authors:** Ju Young Hong, Seungcheol Ko, Ki Sub Sung, Min Jae Oh, Min Ji Kim, Jung Woo Lee, Yoo Seok Park, Yong Hyun Kim, Joon Sang Lee

**Affiliations:** 1 Emergency Medicine, Yonsei University College of Medicine, Seoul 03722, Republic of Korea; juyoungbaby@yuhs.ac (J.Y.H.);; 2 School of Mechanical Engineering, Yonsei University, Seoul 03722, Republic of Korea; 3 SS-ENG Co., Ltd., Bucheon 14449, Republic of Korea; 4 AI & Energy Research Center, Korea Photonics Technology Institute, Gwangju 61007, Republic of Korea

**Keywords:** respiratory infection, computational fluid dynamic, intubation, aerosol transmission

## Abstract

The COVID-19 pandemic has highlighted the significant infection risks posed by aerosol-generating procedures (AGPs), such as intubation and cardiopulmonary resuscitation (CPR). Despite existing protective measures, high-risk environments like these require more effective safety solutions. In response, our research team has focused on developing a novel respiratory barrier enclosure designed to enhance the safety of healthcare workers and patients during AGPs. We developed a hood that covers the patient’s respiratory area, incorporating a negative pressure system to contain aerosols. Using computational fluid dynamics (CFD) analysis, we optimized the hood’s design and adjusted the negative pressure levels based on simulations of droplet dispersion. To test the design, Polyalphaolefin (PAO) particles were generated inside the hood, and leakage was measured every 10 s for 90 s. The open side of the hood was divided into nine sections for consistent leakage measurements, and a standardized structure was implemented to ensure accuracy. Our target was to maintain a leakage rate of less than 0.3%, in line with established filter-testing criteria. Through iterative improvements based on leakage rates and intubation efficiency, we achieved significant results. Despite reducing the hood’s size, the redesigned enclosure showed a 36.2% reduction in leakage rates and an approximately 3204.6% increase in aerosol extraction efficiency in simulations. The modified hood, even in an open configuration, maintained a droplet leakage rate of less than 0.3%. These findings demonstrate the potential of a CFD-guided design in developing respiratory barriers that effectively reduce aerosol transmission risks during high-risk medical procedures. This approach not only improves the safety of both patients and healthcare providers but also provides a scalable solution for safer execution of AGPs in various healthcare settings.

## 1. Introduction

Current guidelines for aerosol-transmitted diseases, such as COVID-19, typically recommend placing the patient in an airborne infection isolation room (AIIR) and ensuring that healthcare workers (HCWs) wear personal protective equipment (PPE) such as an N95 Respirator, a gown, gloves, an eye shield to minimize the spread of infection [1]. While placing patients in an AIIR can prevent cross-contamination to other patients, it does not fully protect HCWs who remain within the AIIR. Despite the use of PPE, approximately 5–10% of HCWs who treated COVID-19 patients reported being infected with the virus [2]. Additionally, the extended use of PPE during long shifts can result in significant physical and mental fatigue [2,3].

Saito et al. reported that aerosol-generating procedures (AGPs), including intubation and cardiopulmonary resuscitation (CPR), elevate the risk of respiratory infectious diseases such as COVID-19 [4]. To reduce this risk, healthcare workers have been compelled to develop alternative methods of protection. Shield boxes, also known as aerosol boxes, respiratory barrier enclosures, or intubation boxes, have been developed to contain droplets generated during AGPs [5,6,7]. However, research on these newly developed respiratory barriers has yielded mixed outcomes; while they may reduce droplet spread, their design limitations can hinder procedural efficiency and potentially increase the risk of contamination during procedures [7,8,9].

In particular, several recent studies have investigated the effectiveness of barrier enclosures using both experimental setups and CFD models. For example, Perella et al. used CFD to simulate aerosol and droplet spread during aerosol-generating procedures and found that using a protective shield prevented the escape of up to 100% of particles under optimal conditions. However, incomplete sealing or insufficient suction led to particle escape in worst-case scenarios [10]. The other example, a study comparing acrylic boxes and plastic sheets during extubation procedures, reported that acrylic boxes were more effective in reducing droplet contamination, but practical issues such as cleaning and design inconsistencies were highlighted as potential challenges [11]. While these studies demonstrate the potential of barrier devices in minimizing environmental contamination, they also underscore the need for further optimization and standardized testing methods to ensure their efficacy across various clinical scenarios.

Since 2020, our research team, which includes board-certified emergency physicians and mechanical engineers, has been developing a novel respiratory barrier enclosure with a negative pressure generator that includes a pressure differential detector. The purpose of this device is to isolate the patient’s respiratory area during medical procedures. A key feature of this novel enclosure is a double-layered membrane system that seals the patient’s chest area and can easily be converted into a system-wide isolator, resembling a negative pressure isolation chamber. The intubation hood, which covers the patient’s head and neck, has four access orifices for healthcare workers [12,13].

Our team has reported the efficacy of this newly developed respiratory barrier enclosure in containing droplets during procedures such as intubation and CPR. We also assessed the efficiency of procedures like endotracheal intubation through simulation studies. Although procedural times showed no statistically significant difference after 10 min of adequate practice, participants reported discomfort during intubation due to the endotracheal tube frequently touching the ceiling of the hood [13]. While enlarging the hood size could alleviate this discomfort, our team restricted the size of the intubation hood to ensure it remains useful for imaging procedures, including CT scans and coronary angiography. Based on these findings, we concluded that further work is needed to improve both efficacy and efficiency. Additionally, our preliminary studies indicated a lack of established standard methods for accurately testing the barrier effectiveness of such chambers.

Furthermore, our preliminary studies [12,13] identified the absence of an established standard method to accurately test the barrier effectiveness of enclosures like ours. In response, this study uses computational fluid dynamics (CFD) to simulate droplet behavior and predict potential external leaks. Following design modifications aimed at improving user comfort and reducing droplet leakage, we constructed a new hood and performed real-time droplet leakage tests using the PAO particle leakage method under various conditions. Our results suggest that even a shorter hood designed for comfort can significantly enhance the protective effect of the enclosure, and the absence of a double-layered membrane did not result in substantial droplet leakage, even under rigorous conditions.

## 2. Materials and Methods

### 2.1. Design of Intubation Hood

The intubation hood was designed with dimensions constrained about 500 mm in width, length, and height, each with four patient access orifices. The intubation hood is intended to cover the patient’s head and neck area, shielding it from the surrounding environment. The design and layout of the isolation hood were developed using Autodesk AutoCAD LT 2020 (Autodesk, Inc., San Francisco, CA, USA). The placement of the patient access orifices was determined by assessing the optimal elbow height for healthcare workers during procedures such as intubation or bag-valve-mask ventilation.

**Figure 1:**
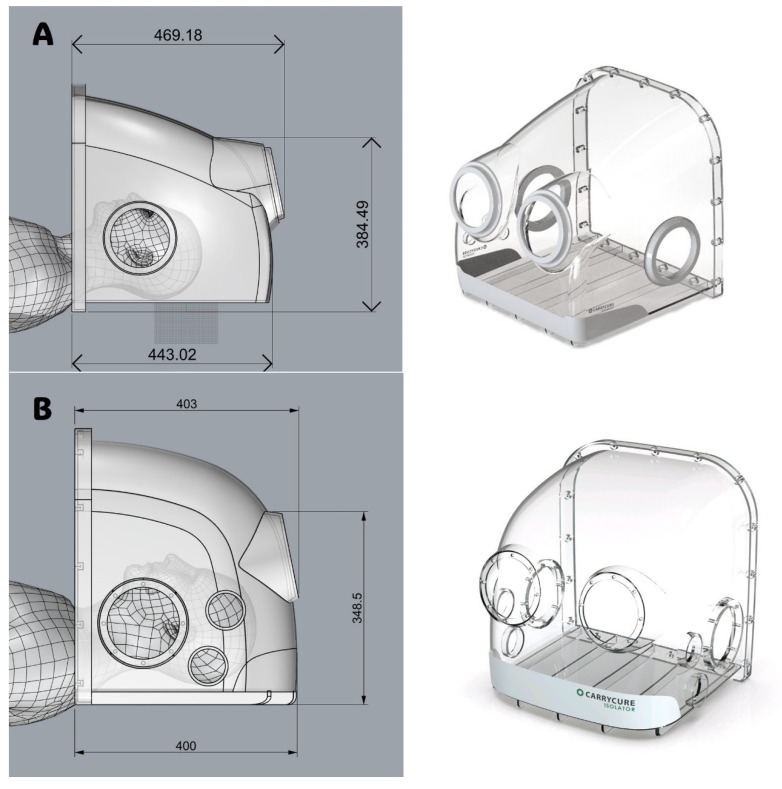
The intubation hood used in in the previous study (**A**) and the modified, shorter version (**B**). In the modified intubation hood, the slope was removed, and ceiling curvature was applied to create more space for procedures.

The model used in the previous study [12] (Figure 1A) revealed that the tip of the intubation tube frequently touched the hood’s ceiling, impeding the procedure [13]. To address this issue and enhance usability for HCWs, the overall length of the hood was reduced. Concurrently, the height of the central portion of the hood was increased by modifying the ceiling curvature (Figure 1B). These adjustments were guided and validated through CFD simulations to ensure that the alterations did not compromise the hood’s barrier performance.

### 2.2. Computational Fluid Dynamic Analysis

The system of equations was solved using ANSYS CFX, Release 2023 R1, commercial flow simulation software. The turbulence model used a shear stress transport model based on the Reynolds-averaged Navier–Stokes equation k−ω, which is excellent for predicting the adverse pressure gradient. The model solves a total of four transport equations: a continuous equation, a momentum transport equation based on the Reynolds-averaged equations, and two for the turbulent kinetic energy, k, and the turbulent frequency, ω. The stress tensor is calculated from the vorticity–viscosity concept.

$$\frac{\partial \rho }{\partial t}+\frac{\partial }{\partial x_{j}}\left(\rho U_{j}\right)=0$$

$$\frac{\partial \rho U_{i}}{\partial t}+\frac{\partial }{\partial x_{j}}\left(\rho U_{i}U_{j}\right)=-\frac{\partial p}{\partial x_{i}}+\frac{\partial }{\partial x_{j}}\left(\tau_{ij}-\rho \bar{u_{i}u_{j}}\right)+S_{P}$$

$$\frac{\partial \left(\rho k\right)}{\partial t}+\frac{\partial }{\partial x_{j}}\left(\rho U_{j}k\right)=\frac{\partial }{\partial x_{j}}\left[\left(\mu +\frac{\mu_{t}}{\sigma_{k}}\right)\frac{\partial k}{\partial x_{j}}\right]+P_{k}-\beta^{\prime }\rho k\omega +P_{kb}$$

$$\frac{\partial \left(\rho \omega \right)}{\partial t}+\frac{\partial }{\partial x_{j}}\left(\rho U_{j}\omega \right)=\frac{\partial }{\partial x_{j}}\left[\left(\mu +\frac{\mu_{t}}{\sigma_{\omega }}\right)\frac{\partial \omega }{\partial x_{j}}\right]+\alpha \frac{\omega }{k}P_{k}-\beta \rho \omega^{2}+P_{\omega b}$$

In addition to the independent variables, the density, ρ, and the velocity vector, U, are treated as known quantities from the Navier–Stokes method. P_k_ is the production rate of turbulence, which is calculated as in the k-ε model, τ is the molecular stress tensor, and S_P_ is the source term of momentum by particle. The model constants are given by: $\beta^{\prime }=0.09$, α=5/9, β=0.075, $\sigma_{k}=2$, $\sigma_{\omega }=2$. $\mu_{t}$ is the turbulent eddy viscosity which is calculated by:

$$\mu_{t}=\frac{\rho a_{l}k}{\max\left(a_{l}\omega ,SF_{2}\right)}$$

Again, F2 is a blending function which restricts the limiter to the wall boundary layer, as the underlying assumptions are not correct for free shear flows. S is an invariant measure of the strain rate.

The unknown Reynolds stress tensor, $\rho \bar{u_{i}u_{j}}$, is calculated from:

$$-\rho \bar{u_{i}u_{j}}=\mu_{t}\left(\frac{\partial U_{i}}{\partial x_{j}}+\frac{\partial U_{j}}{\partial x_{i}}\right)-\frac{2}{3}\delta_{ij}\left(\rho k+\mu_{t}\frac{\partial U_{k}}{\partial x_{k}}\right)$$

Just as the fluid influences the behavior of the particles through forces such as convective heat transfer, there is an opposing effect of the particles on the fluid, which is known as coupling. In this study, we simulate two-way coupling, which is the case where the particles also influence the fluid behavior. To use two-way coupling, the fluid momentum equation must include a particle source term. The momentum source is due to the drag, and the particle source term is generated for each particle as the flow is tracked. The particle source is applied to the control volume where the particles are located during the timestep. The particle source for the fluid momentum equation is obtained by solving the transport equation for the source. A typical equation for a particle source is:

$$\frac{dS_{P}}{dt}=C_{S}\phi_{P}+R_{S}$$

where C_S_ϕ_P_ is the contribution of the particle that is linear in the solution variables and R_S_ includes all other contributions, including the mass transfer term $-\left(dm_{P}/dt\right)\phi_{P}$ where appropriate. This equation has the same form as general particle transport and can be solved in the same way as described above. The source to be added to the continuum is then multiplied by the particle flow rate, which is the mass flow rate of that particle divided by the mass of the particle. In CFX, the particle source term is recalculated each time a particle is injected. The source term is then kept in memory so that it can be applied each time a fluid coefficient is calculated. This allows the particle source to be applied even if no particles are injected in the current flow calculation.

Consider a discrete particle traveling in a continuous fluid medium. The forces acting on the particle that affect the particle acceleration are due to the difference in velocity between the particle and the fluid, as well as to the displacement of the fluid by the particle. The equation of motion for such a particle was derived by Basset, Boussinesq and Oseen for a rotating reference frame, where $m_{p}$ is the particle mass and $U_{p}$ is the particle velocity:

$$m_{p}\frac{dU_{p}}{dt}=F_{D}+F_{B}+F_{R}+F_{VM}+F_{p}$$

which has the following forces on the right-hand side:

$F_{D}$: drag force acting on the particle
$F_{B}$: buoyancy force due to gravity
$F_{R}$: forces due to domain rotation (centripetal and Coriolis forces)
$F_{VM}$: virtual (or added) mass force. This is the force to accelerate the virtual mass of the fluid in the volume occupied by the particle. This term is important when the displaced fluid mass exceeds the particle mass, such as in the motion of bubbles.
$F_{P}$: pressure gradient force. This is the force applied on the particle due to the pressure gradient in the fluid surrounding the particle caused by fluid acceleration. It is only significant when the fluid density is comparable to or greater than the particle density.

The aerodynamic drag force on a particle is proportional to the slip velocity, $U_{S}$, between the particle and the fluid velocity:

$$F_{D}=\frac{1}{2}C_{D}\rho_{F}\frac{\pi d_{P}^{2}}{4}\left|U_{s}\right|U_{S}=\frac{1}{2}C_{D}\rho_{F}\frac{\pi d_{P}^{2}}{4}\left|U_{F}-U_{P}\right|\left(U_{F}-U_{P}\right)$$

where $C_{D}$ is the drag coefficient and $d_{P}$ is the particle diameter; where $C_{D}$ is the drag coefficient and $d_{P}$ is the particle diameter. The drag coefficient, $C_{D}$, is introduced to account for experimental results on the viscous drag of a solid sphere and a value of 0.44 was used in this study.

The buoyancy force is the force on a particle immersed in a fluid. The buoyancy force is equal to the weight of the displaced fluid and is given by

$$F_{B}=\frac{\pi }{6}d_{P}^{3}\left(\rho_{P}-\rho_{F}\right)g$$

where g is the gravity vector, $\rho_{P}$ is the particle density, and $\rho_{F}$ is the fluid density.

In a rotating frame of reference, the rotation term is an intrinsic part of the acceleration and is the sum of Coriolis and centripetal forces:

$$F_{R}=m_{p}\left(-2\Omega \times U_{P}-\Omega \times \Omega \times r_{P}\right)$$

This force is caused by the fact that the particle has to accelerate some of the surrounding fluid, leading to an additional drag of the following form:

$$F_{VM}=\frac{C_{VM}}{2}m_{F}\left(\frac{dU_{F}}{dt}-\frac{dU_{P}}{dt}\right)$$

If the virtual mass force is included, the coefficient $C_{VM}$ is normally set to 1.

The pressure gradient force results from the local fluid pressure gradient around the particle and is defined as:

$$F_{P}=-\frac{m_{F}}{\rho_{F}}\nabla p$$

**Figure 2:**
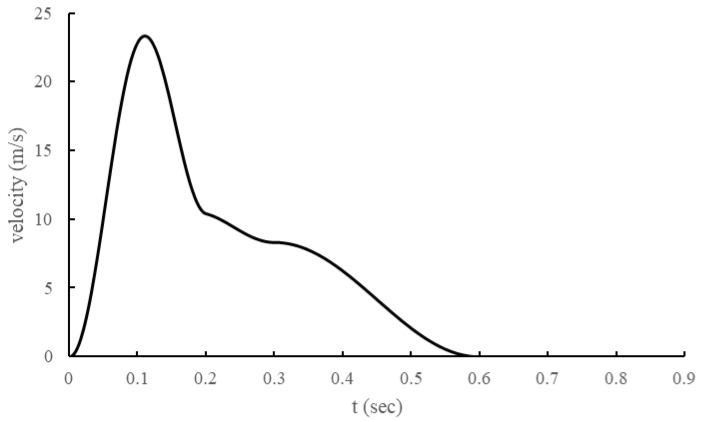
J. K. Gupta et al. inlet velocity profile at the mouth due to coughing [14].

The inlet condition, cough, was modeled as shown in Figure 2 by adapting the model of J. K. Gupta et al. [14]. In addition, the cough droplet size distribution was modeled using the Rosin–Rammler model based on the measurement results of Yang, Shinhao, et al. [15].

Each droplet was subjected to a particle break model, where the model used was the Taylor Analogy Breakup (TAB) model. The time-dependent particle distortion equation according to the TAB model is as follows:

$$\begin{array}{c} y\left(t\right)={We}_{C}+e^{-\frac{t}{t_{D}}}\left[-{We}_{C}\cos\omega t-\frac{{We}_{C}}{\omega t_{D}}\sin\omega t\right]\\ t_{D}=\frac{2\rho_{P}r^{2}}{C_{d}\mu_{P}}\\ \omega^{2}=\frac{C_{k}\sigma }{\rho_{P}r^{3}}-\frac{1}{t_{d}^{2}}\\ We_{C}=We\frac{C_{f}}{C_{k}C_{b}} \end{array}$$

Here, we used 0.5 for the critical amplitude coefficient, $C_{b}$, 5.0 for the damping coefficient, $C_{d}$, 1/3 for the external force coefficient, $C_{f}$, 8.0 for the restoring force coefficient, $C_{k}$, 1.0 for the new droplet velocity factor, and $C_{\nu }$, 10/3 for the energy ratio factor, K.

**Figure 3:**
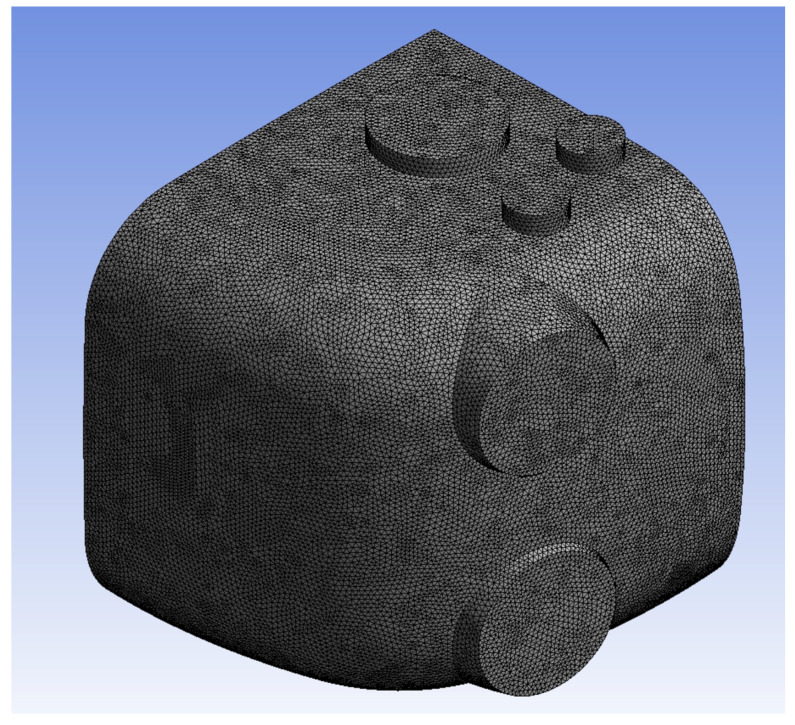
Unstructured mesh of the chamber.

**Figure 4:**
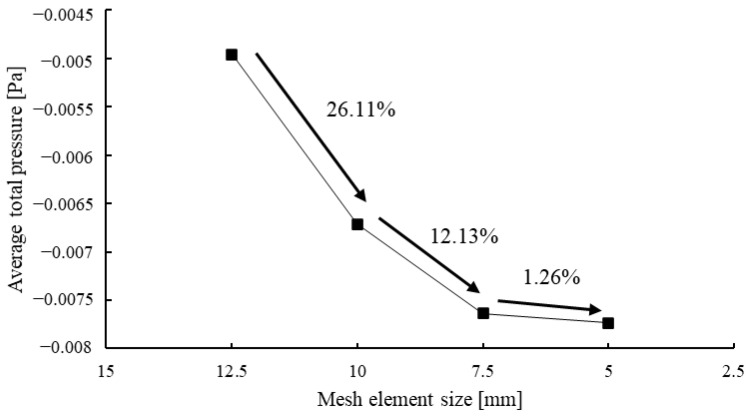
Mesh independence test by using average total pressure.

For the meshes, we set the element size to 5 mm for a total mesh count of 3,780,013, which can be seen in Figure 3. A grid independence test was conducted to determine the sensitivity of the solution to the mesh element size, as in Figure 3. The test results, as depicted in the graph (Figure 4), show the average total pressure variation at different mesh element sizes. As the mesh size decreases from 12.5 mm to 5 mm, the total pressure becomes more stable. Specifically, reducing the mesh size from 12.5 mm to 10 mm results in a 26.11% change in the average total pressure. A further reduction from 10 mm to 7.5 mm yields a 12.13% variation, and finally, decreasing the size from 7.5 mm to 5 mm leads to only a 1.26% change. This indicates that beyond the mesh size of 7.5 mm, the solution becomes nearly independent of the mesh resolution, confirming that the mesh size of 5 mm is sufficient for an accurate solution with minimal computational cost.

### 2.3. Negative Pressure Generator (NPG)

**Figure 5:**
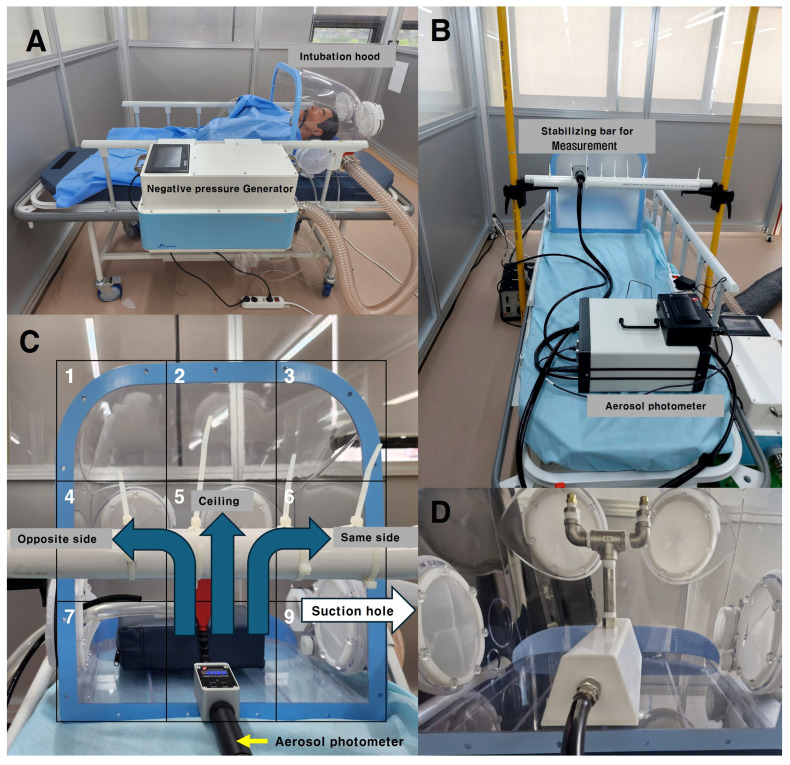
(**A**) An example of an intubation hood and a negative pressure generator with a patient. (**B**) An aerosol photometer was used to measure particle leakage from the intubation hood; a stabilizing bar was used to ensure consistent measurement locations for the detector. (**C**) The open area of the intubation hood was divided into nine sections, and PAO particles were dispersed in three directions (indicated by blue arrows); the suction hole was located on the inside right lateral side of the intubation hood (indicated by the white arrow). (**D**) PAO aerosol was dispersed using an aerosol generator with a valve (developed in-house by our research team).

A negative pressure generator was designed to continuously suction air inside the hood through an 80 mm diameter duct, allowing it to pass through a HEPA filter with 99.7% efficiency. Ventilation fans were installed inside the negative pressure generator, designed to operate up to four levels of boosters depending on the negative pressure differential inside and outside of the intubation hood. The system was programmed to activate an additional booster if the pressure differential dropped below −10 Pa, and to deactivate the booster if the pressure differential exceeded −20 Pa. The pressure differential was continuously monitored using a sensor (differential pressure transmitter ©Beck 93 Sensortechnik GmbH, Steinenbronn, Germany) during suction (Figure 5A).

Without the double-layered membrane, the pressure differential between the inside and outside was maintained below −10 Pa, ensuring that all four boosters remained active. When the hood was completely closed, the pressure differential exceeded −80 Pa with all four boosters running. In this study, the hood was kept in an open state, and its barrier efficiency was assessed under conditions with all four boosters running automatically.

### 2.4. PAO Particle Leakage Testing

In this study, we adapted the PAO particle method, commonly used for HEPA filter integrity testing, to assess particle leakage from the inside of a hood to the outside. This method refers to standards such as ISO 14644-3 [16], which ensure precise detection of leaks. PAO particles were generated inside the hood using an aerosol generator, and concentrations were maintained between 100 and 120 µg/L, in contrast to the ISO standard, which recommends concentrations of 20–30 µg/L, to ensure more robust testing conditions.

An aerosol photometer (Model PH-5, Tec Services, Inc., Atlanta, GA, USA) was used to detect leakage, with significant leakage defined as exceeding 0.3% of the particles generated inside the hood, while any leakage above 0.1% was also recorded (Figure 5B).

To identify areas with the highest leakage, the open area of the hood—the patient’s chest side—was divided into nine sections. Measurements were taken nine times at 10 s intervals over 90 s, at a distance of 15 cm from the hood’s open side. Aerosolized PAO particles were dispersed in three different ways: opposite to the suction hole, directly to the middle top of the ceiling, and toward the suction hole. This entire process was repeated five times. These results were then compared CFD analysis to further evaluate the leakage patterns (Figure 5C).

### 2.5. Coughing and Breathing Condition

The PAO particle injection method was adjusted to simulate both coughing and regular breathing by the patient inside the hood. For the coughing scenario, the concentration of PAO particles within the central area of the hood was increased to reach 100–120 µg/L over 90 s. This amount was then released in six bursts, simulating a coughing model. Additionally, a valve (Figure 5D) was used to control the release of the same amount of aerosol, allowing it to disperse slowly over 90 s to simulate particle emission during normal breathing.

Considering the total number of droplets a patient typically exhales—about 7200 particles per liter with a tidal volume of 450 mL per breath at a rate of 15 breaths per minute—the droplets dispersed in the chamber would reach approximately 780 particles/L over 90 s [16]. This corresponds to a PAO particle concentration of less than 0.0001 µg/L, indicating that the simulated particle concentration used in the study was significantly higher than what is typically generated by a person. This ensured a more rigorous test of the system’s performance.

## 3. Results

### 3.1. Validation of CFD Simulation

**Figure 6:**
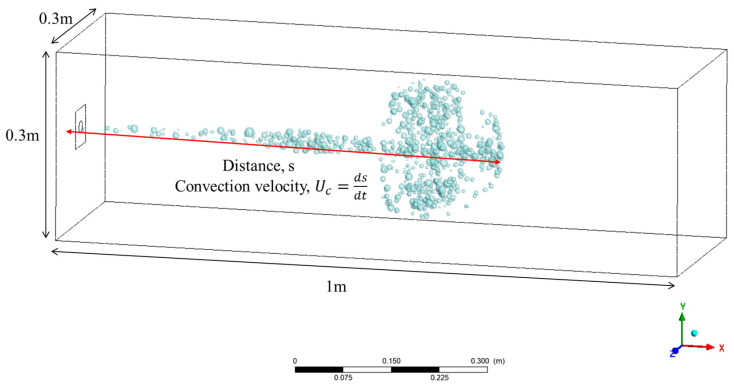
Simulation domain for validation and definition of distance and convection velocity.

**Figure 7:**
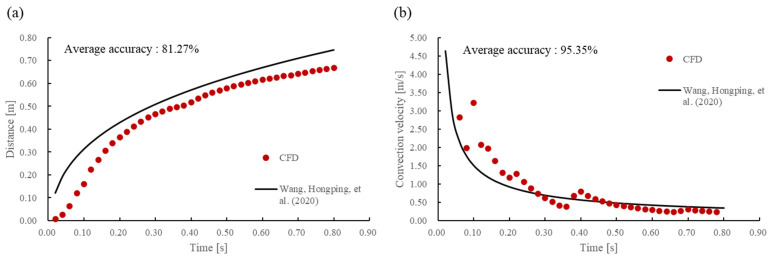
Compare (**a**) distance and (**b**) convection velocity between CFD results and empirical equations (Wang, Hongping, et al. [17]).

Wang, Hongping et al. measured the progression of a cough within a 1 m × 0.3 m × 0.3 m chamber [17]. The cough was designed to be initiated by a human as close as possible to a square opening area of 60 cm. Each experiment was performed by four healthy male volunteers, and each volunteer repeated the experiment three times. The cough and saliva droplet velocities were measured by particle image velocimetry, where the cough process is visualized as a plume of smoke being expelled, as shown in Figure 6, and the horizontal distance from the end of the plume to the cough initiation point is defined as distance, s. Furthermore, the convection velocity is defined as the temporal derivative of s. The experimental data were organized into time functions averaged over 11 cases, and in this study, the CFD model was validated by comparison with the time functions. As shown in Figure 7, the comparison shows high accuracy in the trend and value of the distance, achieving an average accuracy of 81.27% despite the low accuracy due to the initial low absolute value. The convective velocity also shows high accuracy, achieving an average accuracy of 95.35%, indicating that, overall, the CFD model is reliable.

### 3.2. CFD Simulation of Modified Intubation Hood Before Construction

**Figure 8:**
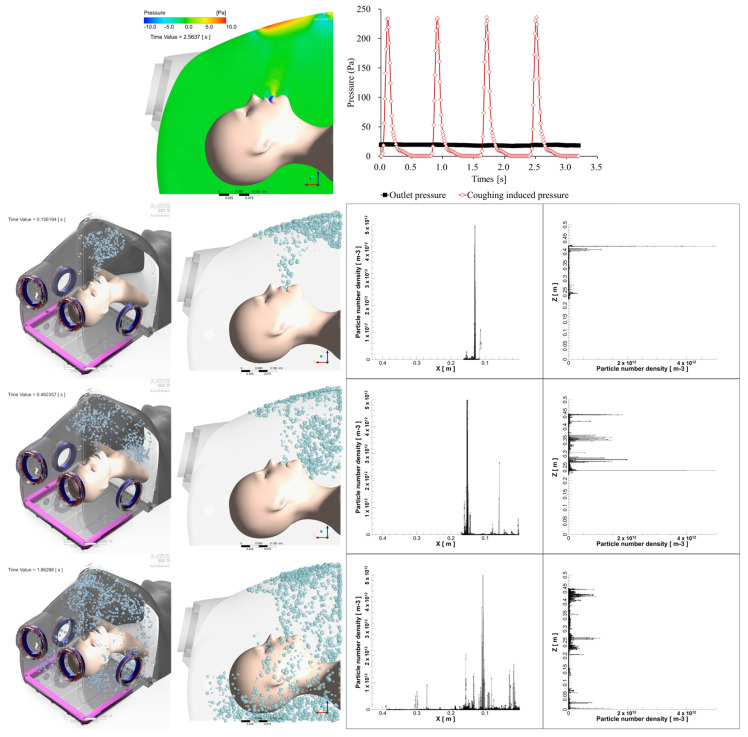
Simulation results of droplet flow showed that the high-pressure region created by the ceiling slope causes droplets to stay near the open portion of the intubation hood, delaying their movement to the suction hole. Additionally, the high pressure generated by coughing exceeds the suction power.

**Figure 9:**
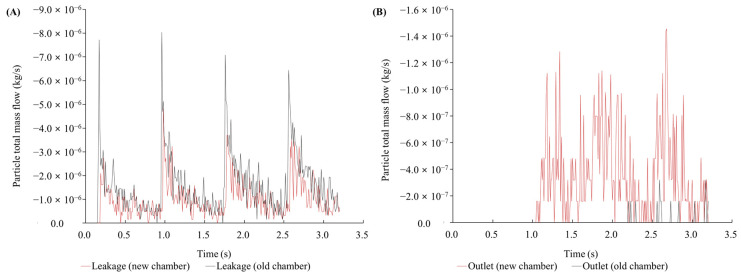
(**A**) The total leakage of droplets decreased by approximately 36.2%, and (**B**) the drain mass flow increased by about 3204.68%.

CFD simulation for the original intubation hood (original hood) was conducted, and the results showed that the slope of the ceiling created a high-pressure area during coughing, causing droplets to flow outside (Figure 8). Based on this result, we removed the slope structure of the original hood and created a curvature to increase the space for procedures and eliminate the high-pressure area (Figure 1). For procedural convenience, the total length of the intubation hood was reduced to 400 mm. A CFD analysis was conducted to compare the efficiency of the original hood with the modified intubation hood (modified hood). The total droplet leakage decreased by 36.2% in the modified hood design, and the droplet drain mass flow increased by 3204.68% (Figure 9).

**Figure 10:**
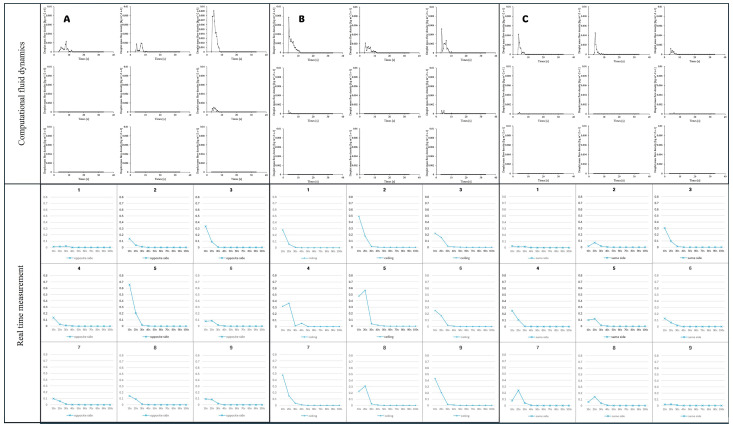
(**A**) CFD simulation of droplet leakage and real time measurement of droplet leakage. (**A**) Opposite side coughing direction. (**B**) Ceiling coughing direction. (**C**) Same side coughing direction.

Using the modified hood design, a simulation analysis of droplet leakage was conducted under three types of droplet spread scenarios with −10 Pa suction power. The open side of the hood was divided into nine sections, and leakage mass flow was measured through simulation analysis. The highest leakage area was observed on the opposite side of the coughing direction (Figure 10A,C) and on both lateral sides in the mid-top spread condition (Figure 10B).

### 3.3. Real-Time Measurement of Droplet Leakage

**Figure 11:**
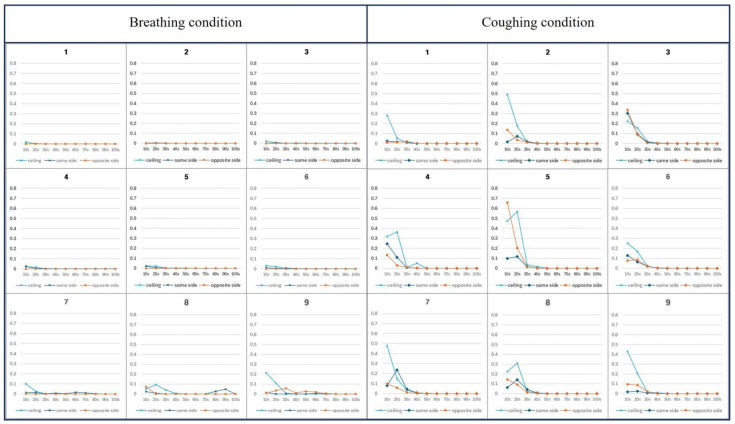
Particle leakage during 90 s from each of the nine sections of the hood. When droplets were spread with high pressure, more particles leaked through the side opposite to the coughing direction. As a result of the CFD simulation, the number of leaked particles was smaller when coughing toward the suction hole direction.

To confirm the safety of the intubation hood with a negative pressure generator, a PAO particle leakage test was conducted under two conditions at 10 s intervals for 90 s. In the breathing condition, which spread PAO particles continuously for 90 s, the maximum leakage amount was less than 0.1%. But in the coughing condition, which spread PAO particles in six bursts over 15 s, the maximum leakage amount was nearly 0.8% (Figure 11).

Different from the CFD simulation results, real-time measurements showed that some particles leaked through the lower section of the hood. When droplets were spread with high pressure, more particles leaked through the side opposite the coughing direction. Similarly, the CFD simulation showed that the number of leaked particles was less when coughing toward the suction hole direction.

**Figure 12:**
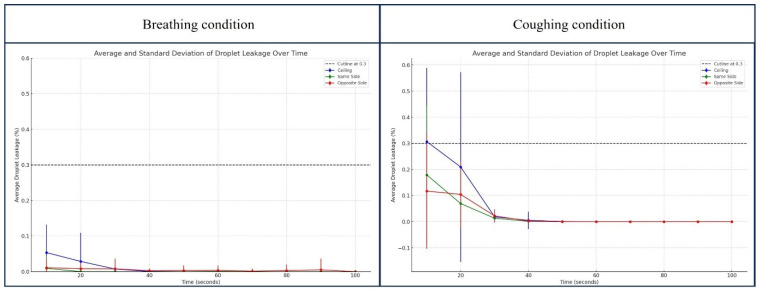
Average and standard deviation of droplet leakage over time. The overall mean percentage of leakage across all sections was less than 0.3%, and the standard deviation of leakage was larger when droplets were spread under higher pressure.

Total leakage in both the breathing and coughing conditions is less than 0.3%, as summarized in Table 1 and shown in Figure 12. The droplet leakage in the breathing condition showed minimal leakage which was largest around 0.0536% at 10 s, and rapidly decreased to nearly zero by 50 s. There is a notable initial spike, but it drops off quickly. In the coughing condition, however, the droplet leakage is significantly higher, with a peak value of 0.3067% at 10 s which gradually drops off as well, but the overall exposure appears higher than during breathing.

## 4. Discussion

Since the onset of the COVID-19 pandemic in 2020, our research team has been developing an intubation hood. Initially, we prioritized the containment efficacy of the intubation hood as the most critical factor, but we encountered challenges in finding an appropriate method to confirm its protective effect [12,13]. Another significant challenge was that increasing the hood’s protective effect by enhancing the fan’s suction power could potentially create more airflow inside the hood, leading to patient discomfort or interfering with their respiration. After much deliberation, our team decided to utilize computational fluid dynamics (CFD) analysis [14,17].

The air inside a small space, such as an intubation hood, exhibits turbulent characteristics due to low viscosity and the perturbations caused by repeated cycles of exhalation and inhalation. Turbulence, in this context, refers to a flow that includes random and chaotic vortices, making an explicit solution impossible, thus necessitating the use of CFD analysis [18,19].

During our preliminary study, CFD analysis indicated that the chamber’s protective effect could be sufficient even without the double-layered membrane of the previously tested novel intubation hood [12,13]. Consequently, in this study, we analyzed the protective effect under open conditions (without the double-layered membrane). We discovered that even if the suction power is sufficiently strong, the airflow inside the hood remains minimal. Our study found that the original intubation hood’s sloped ceiling caused high-pressure areas during coughing, leading to increased droplet leakage. Modifying the hood with a curved design reduced total leakage by 36.2% and improved droplet drainage by 3204.68%. Real-time measurements confirmed that maximum leakage was 0.0536% during breathing and 0.3067% during coughing, with total leakage under 0.3%. The modified hood effectively reduces droplet leakage, enhancing both safety and procedural efficiency.

Perella et al. used CFD to analyze droplet leakage from a hood design that is most similar to our novel hood and predicted that with a suction rate above 160 L/min, leakage would be reduced to almost 0%, even in an open system [10]. In contrast, we conducted our analysis using a lower pressure of 10 Pa, and through design modifications, predicted an improvement in removal efficiency and a reduction in leakage. We also conducted real-time droplet measurements and compared these results with the CFD analysis.

Turer et al. measured leakage using PAO particles and demonstrated that a closed system with an HEPA filter and vacuum machine leaked less than 0.01% of droplets. However, when applied to an open system or with lower suction power, their study reported leakage exceeding 11% [20]. Compared to closed systems, which generate stronger and more concentrated airflow that may have a greater impact on the patient, open systems allow for a larger volume of air to enter but at a lower airflow velocity. This reduced intensity of airflow likely results in less impact on the patient. Additionally, it is more convenient for healthcare workers (HCWs) to perform procedures such as intubation, cardiopulmonary resuscitation, and patient monitoring using an open-type intubation hood, as opposed to a closed-type system.

Simpson et al. found that using aerosol boxes without suction increased particle exposure, particularly during coughing, with larger particles (1.0, 2.5, and 5.0 microns) spreading significantly at 300 s. Similarly, our study observed substantial leakage during the early phase of coughing, but the leakage in our study diminished more rapidly [8]. In this study, without the use of a double-layered membrane, droplet leakage remained below 1% under both coughing and breathing conditions. For comparison, the N95 mask, which is recommended to protect HCWs from respiratory infections during patient treatment, provides a protective efficiency of 95% [1]. Therefore, the protective effect of our novel intubation hood surpasses that of the recommended PPE. If HCWs use this novel intubation hood in conjunction with PPE during aerosol-generating procedures, the overall protective effect would be significantly enhanced.

Developing novel devices like an intubation hood is time-consuming and costly. Modifying the design, size, or conditions, such as suction power or the position of the suction hole, can be expensive and take a long time. This is a significant hurdle in developing such devices. However, using CFD during design and condition changes can be beneficial, as it allows us to find the optimal conditions before physical prototyping. Therefore, we believe that applying CFD in the development and application of such protective equipment will be very helpful, especially since it is already used to assess infection transmission probabilities in indoor environments [16].

We also anticipate that in new pandemic situations involving respiratory infectious diseases like COVID-19, this device could be a solution for overcoming the shortage of negative pressure isolation rooms or facilities, especially during emergency procedures on patients with uncertain infection status, such as angiography, emergency deliveries, emergency endoscopy, or CPR. Indeed, during the COVID-19 pandemic, many emergency procedures were delayed due to uncertainty over the patient’s infection status [21,22,23].

Although droplet leakage varied under coughing conditions and exceeded 0.3%, which is the efficiency threshold for the HEPA filter, our testing conditions were much more rigorous, using particle concentrations 10^5^ times higher than in actual coughing scenarios. Additionally, the density of PAO (0.82–0.86 g/cm^3^) is lower than that of droplets generated during coughing, which are primarily composed of water (1 g/cm^3^). Therefore, we hypothesize that in real scenarios, the actual amount of leakage would be lower than what was observed under our experimental conditions.

The limitations of our study are as follows:

First, the CFD results and real-time measurements do not match exactly. There are differences, particularly in the lower area of the hood, where real-time measurements showed similar leakage to the upper side, whereas the CFD simulation indicated leakage primarily in the upper sections. This discrepancy may be due to the differing characteristics of the droplets in each setup. Further studies using real droplets, similar to those produced by human breathing or coughing, are needed. We are also considering using particle image velocimetry (PIV) to observe the actual movement of droplets and compare these results with the CFD analysis. But replicating real-world coughing conditions remains a challenge. It is difficult to detect evaporative fluids that do not exist under normal air conditions, and ensuring ideal sealing and isolation efficacy is complex. Additionally, using PAO oil in simulations does not perfectly mirror the behavior of real droplets. While we optimized the simulation to closely mimic human coughing, we conducted real-world tests under more extreme conditions with higher aerosol volumes to assess the hood’s performance under stringent circumstances.

Second, the protective effect of the novel intubation hood has not been fully validated under all conditions. Although minimal but significant leaks were observed, our testing conditions were much more rigorous, using particle concentrations 10^5^ times higher than those seen in typical coughing scenarios. Additionally, the density of PAO (0.82–0.86 g/cm^3^) is lower than that of droplets generated during coughing, which are primarily composed of water (1 g/cm^3^). We hypothesize that in real scenarios, the actual leakage would be lower than that observed in our experimental conditions.

Lastly, to implement this intubation hood in clinical practice, further studies are required to prove its protective effect using real microorganisms. Additionally, feedback from HCWs and patients will be necessary to assess its practical applicability. These limitations highlight the importance of CFD optimization before real-world testing to ensure the system performs effectively under stringent conditions.

## 5. Conclusions

We developed a novel intubation hood to protect healthcare workers during aerosol-generating procedures. Through CFD simulations, we improved the design, reducing droplet leakage and minimizing airflow impact on patients. However, discrepancies between CFD and real-time measurements suggest further study is needed using real respiratory droplets. While our rigorous testing showed promising results, the hood’s protective effect in clinical settings remains to be fully validated. Future work will focus on testing with actual microorganisms and assessing usability in real-world medical environments.

## Figures and Tables

**Table 1:** Average and standard deviation of droplet leakage over time.

	Breathing Condition						Coughing Condition					
Time (s)	Ceiling		Same Side		Opposite Side		Ceiling		Same Side		Opposite Side	
	Mean	SD	Mean	SD	Mean	SD	Mean	SD	Mean	SD	Mean	SD
10	0.0536	0.0786	0.0096	0.0350	0.0111	0.0245	0.3067	0.2822	0.1791	0.2668	0.1172	0.2208
20	0.0288	0.0803	0.0001	0.0004	0.0084	0.0193	0.2093	0.3634	0.0694	0.0810	0.1045	0.1277
30	0.0074	0.0253	0.0000	0.0001	0.0081	0.0289	0.0188	0.0170	0.0135	0.0137	0.0222	0.0250
40	0.0007	0.0030	0.0000	0.0001	0.0028	0.0057	0.0052	0.0334	0.0015	0.0019	0.0026	0.0044
50	0.0000	0.0000	0.0000	0.0000	0.0031	0.0144	0.0009	0.0020	0.0002	0.0003	0.0000	0.0000
60	0.0000	0.0000	0.0006	0.0033	0.0037	0.0137	0.0002	0.0004	0.0000	0.0000	0.0000	0.0000
70	0.0000	0.0000	0.0000	0.0002	0.0015	0.0070	0.0000	0.0001	0.0000	0.0000	0.0000	0.0000
80	0.0000	0.0000	0.0000	0.0001	0.0031	0.0167	0.0000	0.0000	0.0000	0.0000	0.0000	0.0000
90	0.0000	0.0000	0.0000	0.0000	0.0053	0.0317	0.0000	0.0000	0.0000	0.0000	0.0000	0.0000
100	0.0000	0.0000	0.0000	0.0000	0.0000	0.0000	0.0000	0.0000	0.0000	0.0000	0.0000	0.0000

## Data Availability

No new data were created or analyzed in this study. Data sharing is not applicable to this article.
